# Supplementary material for: Emergency of Tsallis statistics in fractal networks
Source: PLoS One. 2021 Sep 29;16(9):e0257855. doi: 10.1371/journal.pone.0257855 (PMC8480727; doi:10.1371/journal.pone.0257855)
Supplement: Attachment — Submitted filename: Reviews.docx [file pone.0257855.s001.docx]

The paper studies flux of information in fractal network, the mechanism to represent information spreading and statistical approach based on Tsallis statistics to investigate a scale-free networks. Differential equations also derived to describe information spreading. In general, the paper is interesting, well organized, and mathematically proof. However, the current form of the paper is not ready to reach on the global audience as it has major weakness, and the significant contributions is also not well highlighted. Please see following concerns:

1. The title should describe on overall research work. The current title is too general. It is not clear how the present work could potentially benefit from a wide range of natural and social phenomena.
2. The author should describe deeper for the application of scale-free networks based in every field so that the reader knows their study may be related to fractal networks or scale-free networks.
3. L13: It is stated that scale-free networks and fractal networks are the same. It’s pretty the same but has a slightly different. If necessary, give more examples of the actual case of scale-free and fractal networks to differential between two. The paper in [12] not mainly defined fractal networks as scale-free networks.
4. L29-L36: The field that related to symmetry scale in complex networks is identified. But this is too broad. The author should focus on the example for each area (same in Point 3)
5. L39: Other studies have presented the association of Tsallis Statistics and other statistical analysis in fractal networks. What is the main contribution to this work and different to others?
6. L56. Again, the author stated the equivalent of fractal and scale-free networks. The paper's title stated the scale-free, but throughout the work, the author uses the term fractal. A bit confusing.
7. L86. *fnet* shoud be italic
8. Put example one or two figures of the networks / nodes / connection to illustrate Definition 1.1 until Definition 1.7.
9. L127. Flux of information in a Fractal Network. Is same with the title?
10. Please re-check the equation and description. Some are not in standard form. For example, in L316, it is not usual to write ‘t0 is the instant when the spread of information starts’. What do you mean ‘instant’?
11. L332. Subsection is information diffusion, but the text is focus of optimization of the information spread to increase efficiency and communication.
12. On the other hand, this work may not be a very recent trend as there are many statistical approaches in another modern field, such as deep learning, neural networks, etc. The author should discuss some recent works in neural network or any related field related to this research in terms of the complexity of the networks.
